# Large Scale Patterns of Antimicrofouling Defenses in the Hard Coral *Pocillopora verrucosa* in an Environmental Gradient along the Saudi Arabian Coast of the Red Sea

**DOI:** 10.1371/journal.pone.0106573

**Published:** 2014-12-08

**Authors:** Martin Wahl, Abdulmohsin Al Sofyani, Mahasweta Saha, Inken Kruse, Mark Lenz, Yvonne Sawall

**Affiliations:** 1 Department of Benthic Ecology, GEOMAR Helmholtz Centre for Ocean Research, Kiel, Germany; 2 Department of Marine Biology, King Abdulaziz University, Jeddah, Saudi Arabia; King Abdullah University of Science and Technology, Saudi Arabia

## Abstract

Large scale patterns of ecologically relevant traits may help identify drivers of their variability and conditions beneficial or adverse to the expression of these traits. Antimicrofouling defenses in scleractinian corals regulate the establishment of the associated biofilm as well as the risks of infection. The Saudi Arabian Red Sea coast features a pronounced thermal and nutritional gradient including regions and seasons with potentially stressful conditions to corals. Assessing the patterns of antimicrofouling defenses across the Red Sea may hint at the susceptibility of corals to global change. We investigated microfouling pressure as well as the relative strength of 2 alternative antimicrofouling defenses (chemical antisettlement activity, mucus release) along the pronounced environmental gradient along the Saudi Arabian Red Sea coast in 2 successive years. Microfouling pressure was exceptionally low along most of the coast but sharply increased at the southernmost sites. Mucus release correlated with temperature. Chemical defense tended to anti-correlate with mucus release. As a result, the combined action of mucus release and chemical antimicrofouling defense seemed to warrant sufficient defense against microbes along the entire coast. In the future, however, we expect enhanced energetic strain on corals when warming and/or eutrophication lead to higher bacterial fouling pressure and a shift towards putatively more costly defense by mucus release.

## Introduction

Inter-population variation of important biological traits at medium to large geographical scales may provide information about the genetic and/or environmental mechanisms controlling the expression of this trait [1]. Defense mechanisms against pathogens, parasites, consumers or foulers are selectively relevant traits [2] given the recognized importance of these agents for individual and population fitness. Nonetheless, their geographic variability within a given species is rarely known (but see [3], [4]).

The almost omnipresent threat of fouling may be warded off by a potential host with the help of a variety of mechanisms [5] among which secondary metabolites play a prominent role [6], [7]. Patterns of quantitative variation in secondary metabolites other than anti-foulants at the temporal, spatial and/or population scale have been reported from sponges [8]–[10], soft corals [11], [12], macroalgae [13], [14], tunicates and bryozoans [15]. These studies also made an attempt to provide a possible base for our understanding of the evolution and ecology behind such variations. For instance, the regulation of antifeeding defenses may be controlled by spatial and temporal changes in grazing pressure [16]–[18]. In contrast, triggers for antifouling defense regulation are unknown so far [19].

For the well-being of scleractinian corals, as for many other species relying on a functional body surface, a control of the settlement and spread of beneficial versus pathogenic bacteria and a limitation of microbial and macrobial overgrowth by antifouling defenses is certainly of prime importance [20], [21]. A reduction of antifouling defense in the presence of a fouling threat may lead to uncontrolled overgrowth and/or bacterial infection of the coral [22], [23]. Any non-stochastic variation in the strength of a species' antifouling defense may reflect either a concurrent variation in fouling pressure (if defense is tuned to threat which is unknown so far) or changes in the well-being of the producer [24], [25]. Regarding defense variability some other benthic groups have been investigated slightly better than corals. Seasonal variations in the strength of antimicrofouling defences in macroalgae are reported to co-vary with the abundance of bacteria (potential microfoulers) and possibly light energy (although underwater light was not assessed in the cited studies) [26], [27] but not unambiguously with temporal fluctuations of resources [19], [27]. Plouguerne et al. [28] reported spatial variation in anti-micro and anti-macro fouling activity of the brown seaweed *Sargassum vulgare* collected at five locations separated by 50–100 km along the coast of Rio de Janeiro. Some sponge species show seasonality in their antifouling defenses [29]. If this is not a publication bias, then the fact that the few investigations into the variability of antifouling defences all confirm their existence suggests that defense variability may be a common phenomenon. Knowledge about antifouling defense variability in time or space is ecologically relevant and may inform about control and drivers of defense production. Regrettably, appropriate investigation are not only rare but in most cases whole tissue extracts instead of surface extracts were tested, a procedure rendering a distinction between stored and deployed antifouling compounds impossible [19].

In reef-building corals energy acquisition and calcification depend very heavily on the photosynthesis of symbiotic zooxanthellae [30]. To warrant this vital function, corals maintain their surface free of macrofoulers by two prime mechanisms, mucus secretion (e.g. [31]), and chemical defense [21], [32]. This apparently is achieved by a cooperation of the various components of the coral holobiont: the polyps, the symbiotic dinoflagellates and the associated microbiota (mainly bacteria) [32]).The associated bacteria occur in three different microhabitats: the skeleton, the tissue and – most relevant for this investigation - the surface mucus layer [33]. Based on the amount of photosynthates provided by the zooxanthellae [34], the polyp excretes variable amounts of mucus which serve multiple purposes such as mucociliary feeding or particle (sediment, bacteria) removal [35]. In addition, the mucus layer by its nutritional properties and as a boundary concentration layer for coral secondary (and primary) metabolites represents a microhabitat which favours the establishment of specific bacterial consortia [31], [36]. The composition of these coral-associated bacterial communities seems to be quite specific for a given coral species and similar among geographically separated populations of this species [33]. The surface-bound bacteria, in their turn, may influence further bacterial or eukaryotic fouling by (i) pre-emption of space and resources or (ii) the production of antifouling compounds [21], [25]. The holobiont's combined capacity to ward off pathogens, parasites and macrofoulers is of vital importance to the health of corals and coral reefs. This capacity has been suggested to weaken under elevated temperature [37] permitting the infection by pathogenic bacteria such as *Vibrio coralliilyticus* in *Pocillopora damicornis* [38]. The increasing prevalence of coral disease with rising temperature could be due to a temperature-triggered activation of bacterial virulence or a weakening in the holobiont's antimicrobial defences [38]. It has been suggested that most of the coral “diseases” may factually constitute a poly-strain invasion of a stress-weakened host by formerly associated bacterial strains [22], [39]. Another hypothesis postulates that elevated temperature enhances pathogen prevalence [40], [41]. Any investigation of the chemical fouling-control of the coral holobiont should include three components: the outer coral surface, its mucus cover and the associated bacteria.

In order to assess the relative importance of antimicrobial defense versus mucus production and their possible regulation, a simultaneous assessment of the two processes was conducted in an important and wide-spread scleractinian holobiont along the natural environmental gradient of the Red Sea. The present study is one of the first attempts to describe large spatial patterns of defenses in the important reef building coral of the Red Sea and Indian Ocean, *Pocillopora verrucosa*. In an effort to screen for potential causes of such variation we evaluate relationships between the strength of chemical and/or mucus defense and environmental and biological variables such as temperature, nutrients and microbial fouling pressure.

This part of the Red Sea is still in an apparently healthy state and only at a handful of major towns (e.g. Jeddah) or industrial agglomerations represent instances of direct anthropogenic impact [42], [43]. The man-made disturbances or pressures mainly consist in coastal constructions (direct damage to the reefs, increased sedimentation during construction, modification of current regimes), industrial pollutants (oil, other chemicals, wastes) or increased fishing pressures and waste water inflow close to larger cities. None of these disturbances is quantified or monitored to our knowledge. Outside these restricted zones of anthropogenic impact, the coastal coral reefs are little altered. At the same time, this extensive reef system covers a wide gradient of environmental factors such as nutrient load, salinity and temperature [43]. From south to north along these 1600 km of Saudi Arabian coastline exists a gradient of decreasing nutrients and productivity (a 2-5 fold decrease in nutrients and a more than 10-fold decrease in surface chlorophyll a), of decreasing mean annual temperature by 5°C and of increasing mean salinity by 3 psu. Details of this multiple environmental gradient are given elsewhere [42], [43]. All these features make the fringing reefs along the Saudi Arabian Red Sea coast a rewarding target for the investigation of present and future stresses.

We hypothesize that if large scale variation in coral antimicrofouling defences exists along the Saudi Arabian Red Sea reefs it might relate to variations in (i) microfouling pressure, (ii) resource availability and/or (iii) elevated temperature. Since this was the first observational study of its kind in the Saudi Red Sea we focussed on the detection of patterns and did not attempt to identify chemical structures or to unequivocally point out drivers for variation. However, the detected correlations may hint at mechanistic relationships in some of the pairings and may help focussing follow-up investigations.

## Material and Methods

### Study sites

**Figure 1:**
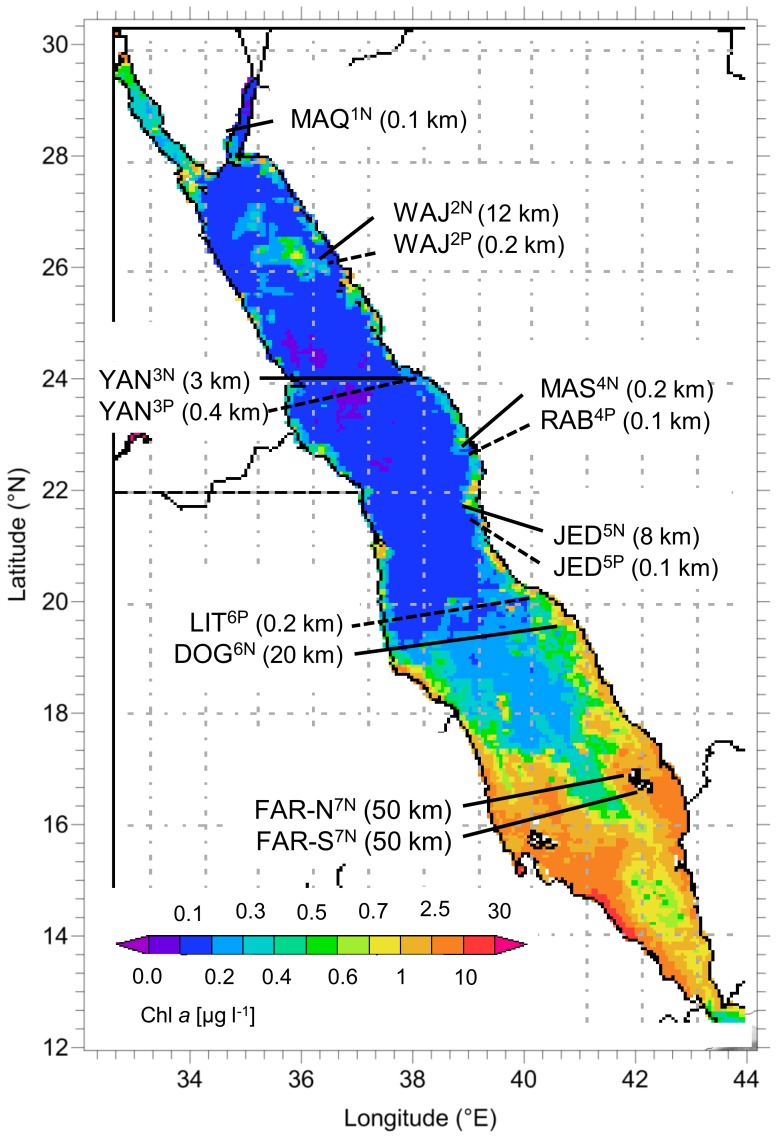
Map of the Red Sea showing (A) the sampling sites from N to S Maq = Maqna, Waj = Al Wajh, Yan = Yanbu, Mas = Masturah, Rab = Rabigh, Jed = Jeddah, Lit = Al Lith, Dog = Doga, and Far = Farasan Islands (coordinates can be found in **Table 1** **) with the mean annual chlorophyll** ***a*** **(Chl** ***a*** **) concentrations from April 2011 to March 2012 at the surface.** Chlorophyll *a* data are derived from satellite images of NASA, Giovanni online data system, Ocean Color Radiometry, data set: MODIS-Aqua 4 km.The superscripts behind the 3-letter site abbreviation designate the site sequence from north to south (digits) and the pollution status with “N” =  non-polluted and “P” =  polluted. The km values indicate the reefs distance from shore.

Corals were collected at 13 locations on the Saudi Arabian Red Sea coast between the Jordanian border in the north and the Yemen border in the south (Fig. 1, Table 1). The sampling sites can be grouped in 7 regions from north to south separated by 130 to 300 km: 1  =  Maqna in the Gulf of Aqaba (“Maq”), 2 =  Al-Wajh (“Waj”), 3 =  Yanbu (“Yan”), 4 =  Rabigh/Mastura (“Mas”), 5 =  Jeddah (“Jed”), 6 =  Farasan Banks (“Dog”), 7 =  Farasan Islands (“Far”). Superscripts added to these site abbreviations in the text, tables and graphs designate the sites' relative position from North to South (numbers) and its pollution status (“N” for non-impacted, “P” for polluted). With the exception of the northern-most and the southern-most sites, in each region an apparently “pristine” and an anthropogenically impacted site were chosen. It should be noted, however, that this classification was done only on circumstantial evidence since no environmental monitoring is done in these regions. More detailed information on the sites is given in table 1 and by Kürten et al. [43] as well as by Sawall et al. [42].

**Table 1:** Investigated sites along the Saudi Red Sea coast.

Station	Impact	N (deg)	E (deg)	Reef type, distance to coast, orientation, slope, notes
**Maqna^1N^**	pristine	28.5253	34.8044	fringing reef, 100 m, W, moderate slope to -18 m.
**Al Wajh ^2P^**	nearly pristine, desalination plant	26.2417	36.4477	fringing reef, 200 m, WSW, vertical slope to -20 m.
**Al Wajh ^2N^**	pristine	26.0680	36.3567	patch reef, 12 km, W, steep slope to -20 m.
**Yanbu^3P^**	impacted: petro chemical, desalination plant, power plant, coastal constructions.	23.9550	38.2053	fringing reef, 200 m, SW, shallow slope to a max of 10 m, half of the corals dead, much sedimentation.
**Yanbu^3N^**	pristine	23.9480	38.1756	patch reef, 3 km, W, reef flat at 2-3 m and steep slope to ∼10 m.
**Mastura^4N^**	pristine	23.0430	38.7772	fringing reef, 200 m, W, steep reef slope from 2-16 m depth
**Rabigh^4P^**	impacted: power plant, cement plant, desalination plant.	22.6260	39.0414	fringing reef, 100 m, WSW, steep slope to 10 m, heavy sedimentation, most corals dead
**Jeddah^5N^**	pristine	21.7530	38.9627	patch reef, 8 km, W, shallow slope to -15 m
**Jeddah^5P^**	impacted, city run off, sedimentation	21.5940	39.1047	fringing reef, 100 m, W, steep slope to -15 m
**Lith^6N^**	semi pristine, protected area	20.2394	40.0080	fringing reef, 500 m, SW, shallow slope
**Lith^6P^**	heavily impacted: waste water outflow of shrimp farm.	20.1475	40.2328	fringe reef, 200 m, S, shallow slope to -10 m, many dead corals, crown-of-thorns
**Doga^6N^**	pristine	19.6141	40.6382	patch reef, 20 km, S, shallow slope to -20 m
**Farassan-N^7N^**	pristine	17.0958	41.9058	fringe reef, 50 km, N, steep slope to -9 m
**Farassan-S^7N^**	semi-pristine	13.5794	42.1494	fringe reef, 50 km, SW, shallow slope to -12 m

Sites from north to south with status of impact, geographical position, and some information about the reef type (more details in [42], [43]. Superscript coding: 1–7 =  site sequence from north to south, “N” =  non-impacted (i.e. pristine), “P” = polluted (i.e. impacted).

### Environmental parameters

All environmental data used in the present paper were collected in two expeditions taking place in February-March 2011 and March 2012, respectively. Temperature was measured continuously with temperature loggers (Hobo Pendant, Onset USA) from February 2011 to March 2012, while nutrients (total phosphorous and nitrogen), chlorophyll a concentration and total carbon were measured in water samples (n = 3) taken during the expeditions in the experimental depth of 5 m at each site. The measurement procedures followed standard protocols and are described elsewhere [42]. Light extinction with depth (K_d_, proxy for turbidity) was calculated with the Lambert-Beer equation [44] and the light intensities (photosynthetic active radiation – PAR) measured at the surface and at 5 m (n = 5) during both expeditions using a PAR sensor (RAMSES-ACC-VIS, Germany). For the evaluation of the relation between temperature and the diverse response variables the regional average temperature over the four weeks preceding the coral sampling was used. The rationale was that the conditions over the preceding weeks affect the condition of the corals at the moment of sampling most intensely.

Microfouling pressure was assessed at each non-polluted site during the 2012 expedition. Six replicate microscope slides covered with Lumox plastic foil (Greiner BIO-ONE GmbH, Frickenhausen, Germany) were exposed in the vicinity (<2 m distance) of the corals in a vertical position at 3 m depth for 48–72 h. Upon retrieval, they were rinsed softly but abundantly with sterile-filtered (0.2 µm) seawater to remove unattached particles and then fixed in 4% formalin until processing. After the return to Kiel, the biofilm attached to the foil was stained with DAPI (4',6-diamidino-2-phenylindole dihydrochloride, Sigma-Aldrich, Germany) for 10 min. Bacteria were counted under an epifluorescence microscope (Carl Zeiss, Germany, Axio Scope.A1) using for excitation a UV-LED at 365 nm (Carl Zeiss) and receiving emission with a long pass filter (Semrock, USA, BrightLine HC 409–800 nm). The bacterial numbers in ten random fields of vision were counted and averaged. Subsequently, cell numbers were standardized by area and duration of in situ exposure to obtain a measure of microfouling pressure expressed as number of cells settling per mm^2^ and hour.

### Study species and sampling design for antimicrofouling assessment

*Pocillopora verrucosa* occurs along the entire coast line. Their relative abundance was higher in the northern and central than in the southern part of the coast [42]. *Pocillopora verrucosa* shows a bushy, stout growth with the cream, brown, pink or bluish colonies rarely exceeding 50 cm height [45]. East-Pacific *P. verrucosa* were described to suffer heat stress at a temperature exceeding 30°C [46]. A decrease of metabolic performance beyond 29°C was described for *P. verrucosa* from French Polynesia [47]. This species reproduces sexually (hermaphrodite broadcast spawner [48]) and genetic mixing (“panmixia”) is assured over substantial distances [49]. Evidence of a decline in performance (calcification) beyond 30°C and an apparent lack of adaptation to regional temperature regime along the Saudi coast are reported by Sawall et al (under review). Other species of the genus have been shown to chemically control bacterial recruitment onto the colony surface [32], [50], [51].

In 2011 at all 13 sites and in 2012 at the 7 “pristine” sites only, we collected by Scuba three replicate samples from coral individuals which were at a minimum distance of 10 m from each other and at a depth between 3 and 5 m. The sampling itself was done by breaking off a 8–10 cm long branch from a colony and placing it in a clean zip-lock bag under water. Back on the beach (i.e. within 30 min of collection), the branches were immediately dipped in ethyl-acetate (HPLC grade) for 10 seconds for an extraction of metabolites of a wide polarity range located at the colony surface. This procedure was established and standardized for other organisms [19], [52] but not verified for this coral species. While we cannot exclude that some tissue metabolites were extracted, the vast majority of the extract should stem from the coral surface (i.e. outer polyp epithelium, surface mucus plus associated microbiota). Solvent and extract were kept at 4°C for further processing in Jeddah. The extracted coral fragments were labelled, sun-dried, and stored for later use.

Back in the lab of KAU (Jeddah), the surface of the fragments was determined after the wax-coating technique [53], however we used oil paint instead of wax. Briefly, the fragment was dipped into oil paint twice, while it was dried and weighed after each dipping. The added weight between the first and second dipping was determined and the surface area calculated after the standard curve, which was constructed by coating differently sized wooden cubes of known surface area in the same manner.

### Measurement of mucus release and photosynthetic rate

Mucus release and photosynthesis were measured in March 2012 at the “pristine” sites on a separate set of *Pocillopora* individuals than those used for the chemical extraction. Six coral fragments per site were chiselled off the central part of six coral colonies (one fragment per colony), each glued to a plastic screw, fixed to a support and left for one day in situ for recovery. For in situ mucus release measurements, four transparent acrylic chambers (∼950 ml) equipped with a battery-run stirrer for water mixing and a “window” of Teflon membrane for gas exchange were deployed at the experimental site. Three chambers were equipped with one coral fragment each and one chamber served as a coral-free control. The chambers were filled with surrounding water, closed and corals were incubated from 0900 to 1600 hrs. An initial water sample was collected during the start of the incubations with folding-canisters from the surrounding of the chambers and final water samples were collected at the end of the incubation period with 1–l bottles from each incubation chamber. The water samples were kept in cooler boxes and filtered through pre-weighed GF/F filters in the evening of the same day. Incubations were repeated with the remaining 3 coral fragments on the following day. The filters were dried (60°C) until constant weight and the carbon content was measured with a CN analyzer (Flash 2000, Thermo Scientific, USA, calibrated with Acetanilid) from the initial (n = 3) and final water samples (n = 1 of each chamber). Mucus release was expressed as the differences of carbon between the initial and final samples (minus the control). Only total carbon was measured assuming negligible rates of dissolved mucus release in *Pocillopora* species of the Red Sea [54].

In situ measurements of photosynthetic rates were conducted in parallel to the mucus release measurements with another set of incubation chambers. The measurement procedure and the photosynthetic rates over a daily cycle (photosynthesis-irradiance [P-I] curves) are described by [55].

The daily rates of mucus release are calculated assuming a 12∶12 h day∶night cycle with the measured rates being the day-time rates and 25% of the measured rates being the night-time rates [54]. Daily rates of photosynthesis were calculated from the data of the P-I curves. For this idealistic light curves over the day were reconstructed (light versus time over 24 h), which then were used to assign a photosynthetic rate to each irradiance value. Finally, the reconstructed irradiance intensities and photosynthesis rates were combined to calculate the daily photosynthetic rate. Mucus release and photosynthetic rate were standardized to coral surface area.

### Extract processing

In the lab of KAU the extracts were transferred to pre-weighed glass vials, then the solvent was evaporated under vacuum at 30°C, and the extract dry weight was determined. The extracts were transported to Kiel (Germany) in a dry state for further processing.

In the Kiel lab, extracts were re-dissolved in isopropanol at a concentration such that 1 µl contained the extract amount found on 0.94 mm^2^ of coral surface (isopropanol only in the control wells). This allowed coating the inner surface of the test wells (Greiner 96 well plates) at natural concentration (see settlement bioassay chapter). After running the screening of the site-specific antimicrofouling activity most of the crude extracts were used up. The identification of the bioactive compound(s) in this coral species is planned for a follow-up investigation.

### Isolation of Red Sea bacterial strains

Approximately 1 ml of sediment from 1 m depth in a bay close to the field station of KAU in Obhur (Jeddah) was suspended in 9 ml of sterile sea water. After vigorous shaking for one minute, 1 ml of the resulting suspension was log-diluted in 5 steps. Of each dilution step, 20 µl were spread on solid culture medium in a petri dish (15 g agar agar, 2.5 g peptone, 0.5 g yeast extract in 1 L sea water at local salinity of 35) and incubated for 48 h at 28°C. This procedure was repeated with three replicated sediment samples. From each sediment sample, 1 or 2 well isolated colonies at an appropriate dilution step were sampled, inoculated in liquid medium (same composition but without agar), cultivated for 24 h, log-diluted and spread on solid medium, then picked again. After three such purification runs, clearly distinguishable strains were conserved on 10 replicated Roti-Store cryo vials (Carl Roth GmbH, Karlsruhe, Germany) at −80°C until further usage.

For genotypic identification, the isolated Red Sea strains plus one gram-positive marine strain of our collection (“BA”) at GEOMAR were re-cultured on agar slant. Two of the Red Sea strains could successfully be re-grown and were arbitrarily labeled “R1” and “R5”. DNA was extracted from this cultured material using the RTP Bacteria DNA Mini Kit (Stratec Molecular GmbH, Berlin, Germany). PCR was conducted following Heindl et al. [56] by using Primers 27f and 1492r and resulted in ca. 1500 bp products. Sequencing was performed using Primers 534r, 342f and 790f on a 3730xl DNA Analyzer (ABI/Life Technologies, Grand Island, NY, USA) by the IKMB in Kiel, Germany. Sequences were aligned and manually corrected using Sequencher (GeneCodes, Ann Arbor, USA) and submitted to NCBI under accession numbers KM054691- KM054693. BLAST on the NCBI blastn suite (Nucleotide collection (nr/nt)) resulted for R5 in a 100% match with *Pseudoalteromonas arabiensis*, for BA in a 99% match with *Bacillus* sp. (possibly *B. aquimaris*), and R1 is an unknown species of *Pseudoalteromonas* (with a 23 bp-difference from R5 per 1503 bp).

### Antisettlement assays

The inner wall of wells on a 96-well plate (all black polystyrene, flat bottom, Greiner) were coated with coral extract dissolved in isopropanol at a concentration corresponding to the amount of extract obtained per unit surface area of the coral. To this end, extract corresponding to 94 mm^2^ of coral surface were added in 100 µl isopropanol. This amount of liquid wets 94 mm^2^ of the inner surface of the microwells (bottom plus wall). Subsequently, the solvent was evaporated during 24h in a freeze-drier. Using this natural concentration we assured to only register ecologically relevant activities. The strains R1, R5 and BA were inoculated in liquid medium (as above), grown over night at 28°C, re-inoculated in fresh medium and again grown overnight. Because the culture medium is not DNA-free and, thus, produces a background fluorescence signal after DAPI staining we washed the bacteria prior to use. For this purpose, the bacterial culture suspensions were centrifuged at 4000 rpm for 5 minutes, the supernatant was discarded and the bacterial pellet was re-suspended by vortexing 9 ml of in sterile filtered (0.2 µm) sea water at Red Sea salinity (35). This procedure was repeated three times. Subsequently, the bacterial suspension was adjusted to an optical density (OD) of 0.8 by addition of sterile sea water when required. For the settlement assay, 100 µl of the bacterial suspension were added to each well. The bacteria were allowed to settle for 3 h at 28°C, followed by a 10 min staining with DAPI. Subsequently, the bacterial suspension was removed from the wells by turning them over onto blotting paper, followed by three rinses (100 ml per well) with sterile filtered sea water. The relative abundance of settled bacteria was assessed by measuring fluorescence using a plate reader (Hidex, Turku, Finland) with an excitation wavelength of 350 nm and an emission wavelength of 453–472 nm. Extract auto-fluorescence was assessed in wells with extract but without bacterial settlement after DAPI staining. Natural bacterial settlement was assessed in wells without extract but with suspension of the different bacterial strains after DAPI staining. Activity strength was expressed as Log Effect Ratio for each coral replicate and strain separately, such that D =  log[(F_be_ – F_e_)/F_b_] where D =  defense strength, F_e_ =  fluorescence in wells containing extract only, F_b_ =  fluorescence in wells with bacteria only, F_be_ =  fluorescence in wells with bacteria and extract. Expressed thus, a value of 0 means that bacteria settle equally well in the absence and in the presence of a given extract (i.e. the extracts were neither repellent or toxic, nor attractive for the strains), a value of -1 means that a given extract reduces bacterial settlement 10-fold and a value of +1 means that a given extract enhances bacterial settlement by a factor 10.

### Statistical analysis

Possible relationships among environmental variables (temperature, total carbon, chlorophyll a, light attenuation, total nitrogen, total phosphorus) were explored by the correlation matrix module in Statistica. Differences among sites regarding microfouling pressure were analysed by a one-factorial ANOVA. Differences in defense strength between “pristine” and “impacted” sites were analysed by t-tests. The relationships between nutrients, temperature, light attenuation and microfouling pressure as supposed “drivers” for the health of corals, sites as the grouping variable and the biological “response” variables mucus production, maximal primary production, and antisettlement defense were explored using distance-based linear modeling (DISTLM) followed by a distance-based redundancy analysis (dbRDA) in PRIMER with PERMANOVA+ extension. The first routine does partitioning of the variation in the resemblance matrix according to a multiple regression model that is suited to elucidate the relationships between a set of environmental “predictor” variables and a multivariate response by the coral. The dbRDA triplot is then based on an ordination of the fitted values that were provided by the regression procedure; in our case including the 4 environmental variables and the tri-variate biological response of the corals. DISTLM also allows running multiple regressions for single responses, which was done to disentangle the relative influences of the 4 environmental variables considered relevant and the 3 coral traits selected. For DISTLM analysis we used as presumed “predictors” the environmental variables total nitrogen (TN) as a proxy for nutrient load, light attenuation (Kd) as an inverse measure for the availability of light energy, temperature, and microfouling pressure as a possible trigger for antimicrobial defense. The remaining environmental variables were ignored since they correlated with at least one of the 4 selected ones. The multivariate coral “response” was composed of the variables productivity of the coral as a proxy for coral performance, chemical antimicrofouling defense and mucus release (as a possibly alternative defense mechanism). Since environmental variables and fouling pressure were only assessed at the “pristine” sites, the “impacted” sites were excluded from these analyses. All the other tests were run in either Statistica or R.

For various analyses and plots raw data were transformed to “z-scores” which are the numerical difference between a datum and the average of the respective data set divided by its standard deviation. This transformation facilitates the comparison of fluctuations of variables irrespective of their scale or unit of measurement [57].

## Results

### Environmental variables

**Figure 2:**
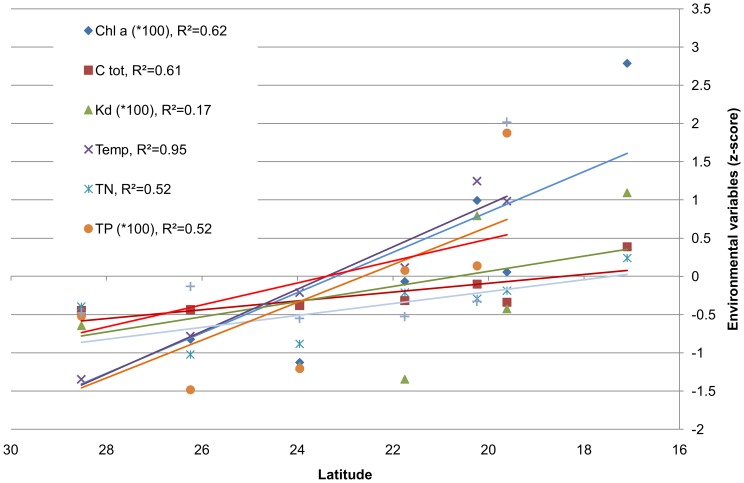
Latitudinal shifts in selected environmental variables along the Saudi Red Sea coast from north to south (non-polluted sites only). Chl a =  chlorophyll a concentration, Ctot  =  total carbon, Kd  =  coefficient of light extinction, Temp  =  temperature, TN  =  total nitrogen, TP  =  total phosphorus, The variables are presented as deviations from their respective all-sites-mean (z-scores). R^2^ values indicate the variance explained by the respective linear model.

We found a multiple N-S gradient in several environmental variables (Fig. 2) all increasing towards the south. The position along the coast (reduced here to the N coordinates since the coast is essentially N-S oriented) explained a substantial part of the variation in chlorophyll a (R^2^ = 62%, p<0.05), total P (R^2^ = 52%, p>0.05), total N (52%, p>0.05), total C (61% P<0.05), and temperature (R^2^ = 92%, p<0.01). Although light attenuation also tends to increase towards the south and microbial fouling pressure sharply rises at the southernmost site, the explained variance of these variables is low (25% or less). Among the environmental variables assessed, chlorophyll a, total nitrogen and total phosphorus are correlated with each other, total carbon correlates with total phosphorus, but temperature and light attenuation do not correlate with any other variable (Table 2). Temperature range during the investigations (both in winter) was from 21°C (N) to 28°C (S).

**Table 2:** Multiple correlations among the environmental variables.

	chla	total C	atten	temp	TN	TP
**chla**		**0.97**	0.68	0.62	**0.85**	**0.96**
**total C**			0.67	0.59	0.74	**0.89**
**atten**				0.25	0.28	0.46
**temp**					0.64	0.69
**TN**						**0.96**
**TP**						

Microbial fouling pressure not included. chla  =  chlorophyll a, total C =  total carbon, atten  =  light attenuation (Kd), temp  =  temperature, TN  =  total nitrogen, TP  =  total phosphorus. Significant correlations (p<0.05) in bold.

**Figure 3:**
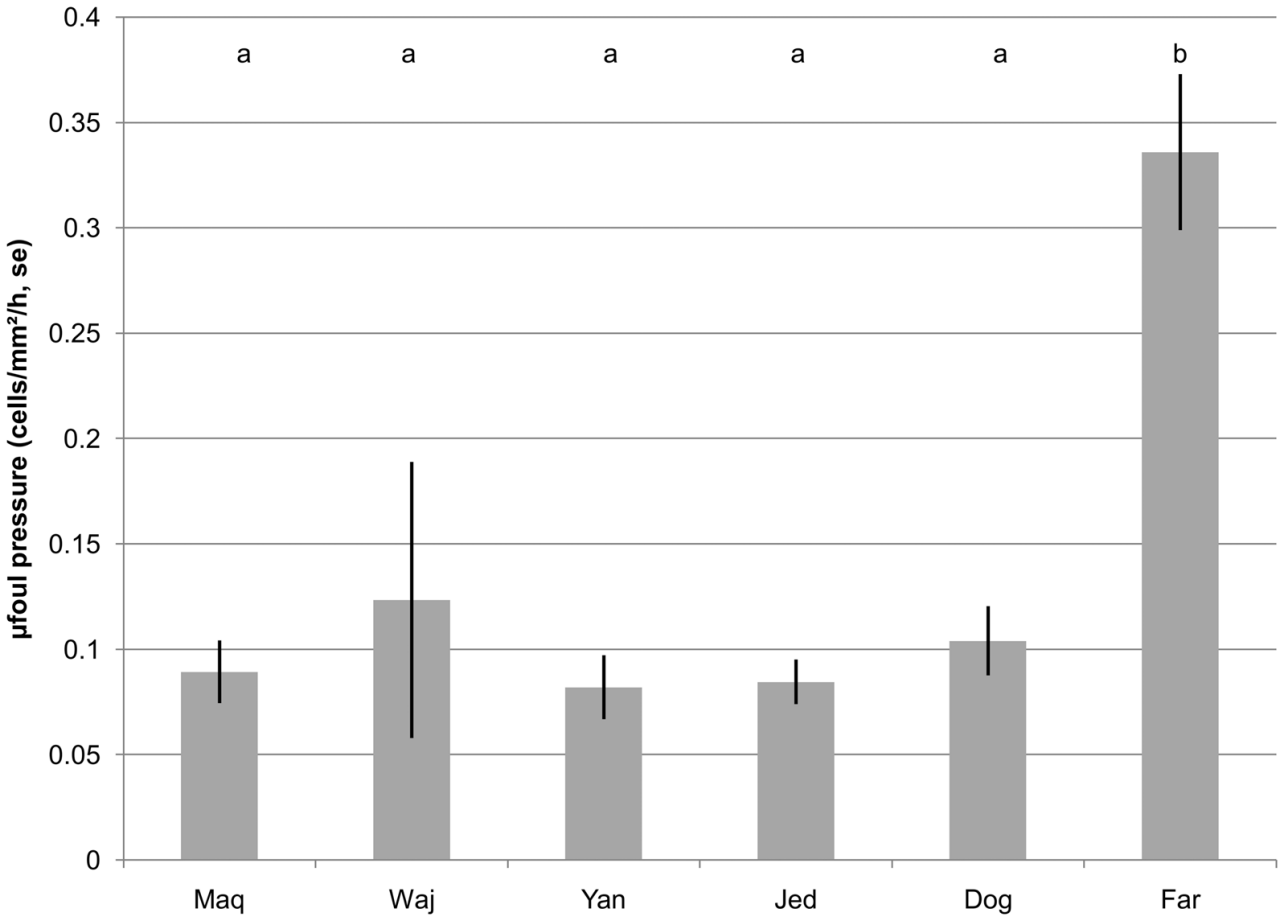
Shifts in microfouling pressure (as settled cells per hour and mm^2^, ± SE) along the Saudi Red Sea coast from north (Maq) to south (Far). All sites sharing a letter in the top of the graph do not differ significantly (see Table 2).

Microfouling pressure, i.e. number of bacterial cells settling per unit surface and unit time measured at the non-polluted sites in 2012, was very low with, on average, only 0.132 bacterial cells settling per hour and mm^2^ (Fig. 3). Only at the southernmost site, Far^7N^, microfouling pressure was substantially higher with 0.34 cells settling per hour and mm^2^ (ANOVA,df = 32, F = 12.9, p<0.001, Table 3).

**Table 3:** Comparison of microfouling pressure among sites.

ANOVA							
	SS	df	MS	F	p		
Intercept	0.583751	1	0.583751	149.8177	0		
Site	0.251714	5	0.050343	12.9203	0.000002		

Tukeys HSD posthoc (Error: Between MSE = .00390, df = 26)							
	Site	Maq	Waj	Yan	Jed	Dog	Far
	**Maq**		0.955813	0.999949	0.999994	0.998745	**0.000138**
	**Waj**			0.904272	0.925038	0.997074	**0.000472**
	**Yan**				1	0.991374	**0.000136**
	**Jed**					0.995142	**0.000137**
	**Dog**						**0.000169**
	**Far**						

ANOVA table. Significant differences (p<0.05) in bold.

Microbial fouling pressure correlates positively with chlorophyll a (part. corr.  = 0.93, p<0.01), but not with temperature (p = 0.47). Relations between microbial fouling pressure and total carbon, TN or TP were not explored since these variables correlate with chlorophyll a.

### Chemical defense of *P. verrucosa*

**Figure 4:**
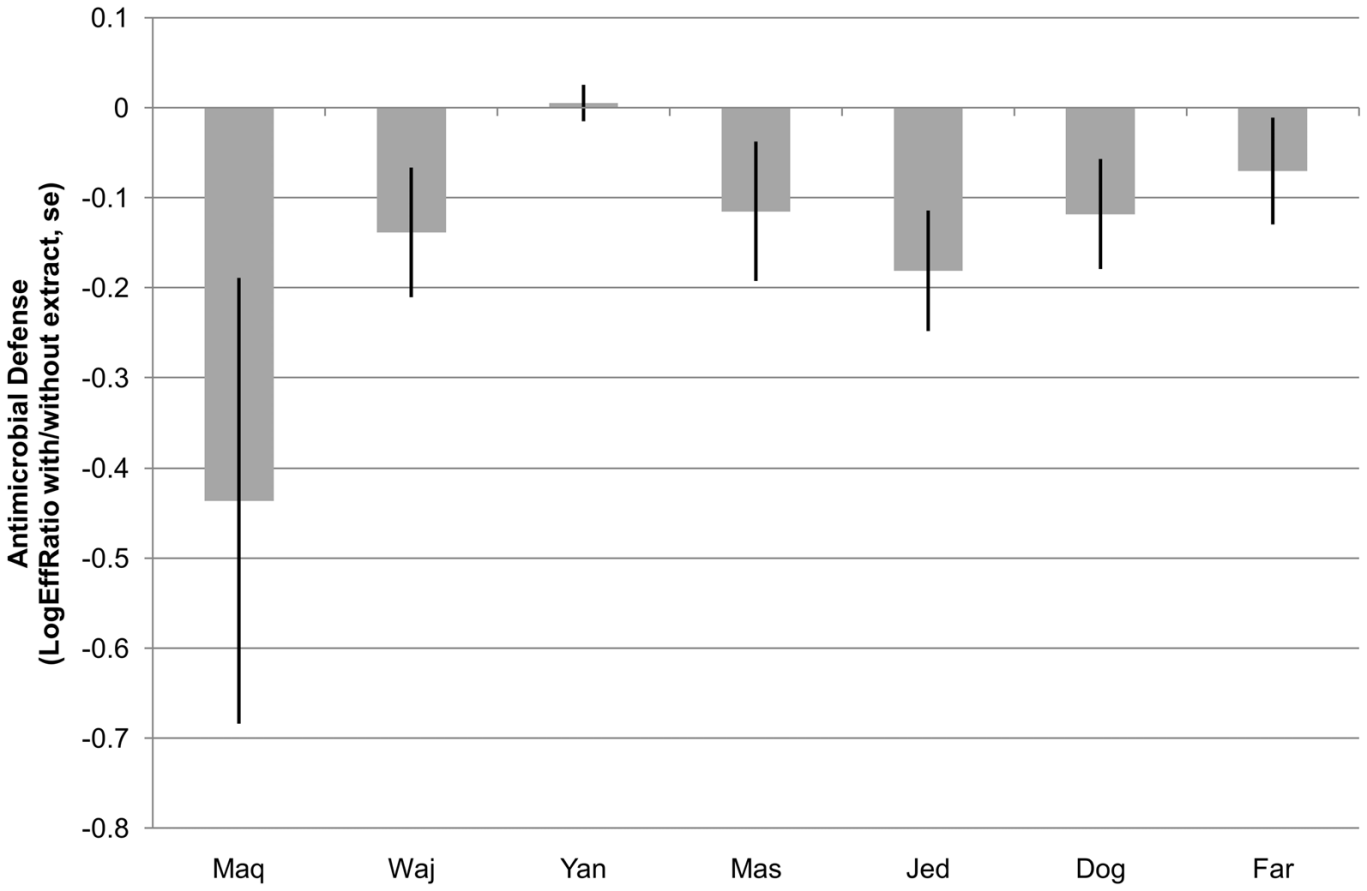
Mean chemical antimicrofouling defense of *P. verrucosa* against three bacterial strains (averaged for the spring samplings in 2011 and 2012) along the Saudi Red Sea coast from north (Maq) to south (Far). Defense strength is expressed as Log Effect Ratio (± SE, see text): more negative values mean stronger repulsion of bacterial settlers by the coral holobiont surface extract.

Corals (i.e. the holobiont surface extracted) at impacted sites were about 20% less well defended against bacterial foulers than their conspecifics at neighboring pristine sites (paired t-test, df = 11, t = −3.6, p = 0.002). To avoid a masking of any potential large scale patterns by this small scale pollution impact, only the results from corals of pristine reefs were used for further analyses. The average defense strength against the target strains showed a quite uniform pattern in the spring seasons of 2011 and 2012. With one exception (Yan^3N^) all populations were chemically defended. Antisettlement activity was strongest in the North (settlement reduction by ca 70%) and decreased in a sinusoidal pattern towards the south (settlement reduction by ca 30%) with an intermediate minimum at Yan^3N^ and an intermediate maximum at Jed^5N^ (Fig. 4)

**Figure 5:**
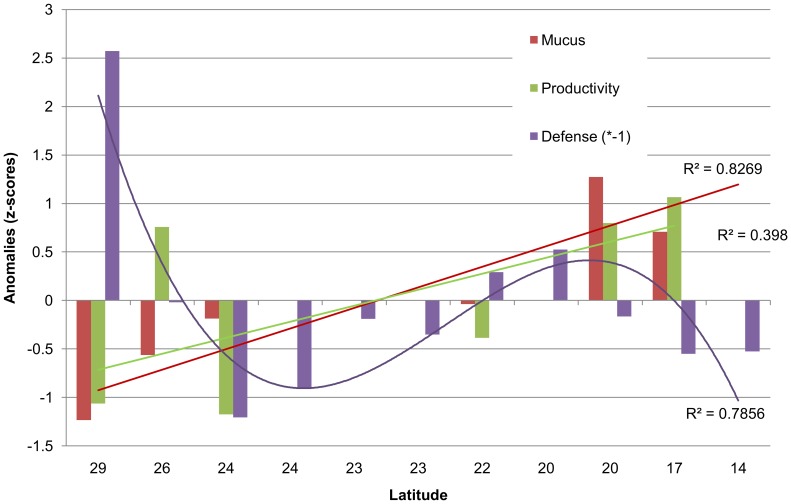
Anomalies (z-scores) of various biological variables (mucus release, productivity, defense strength) along the Saudi Red Sea coast from north (29°N) to south (14°N). R^2^ values is the variation explained by the fitted models.

**Figure 6:**
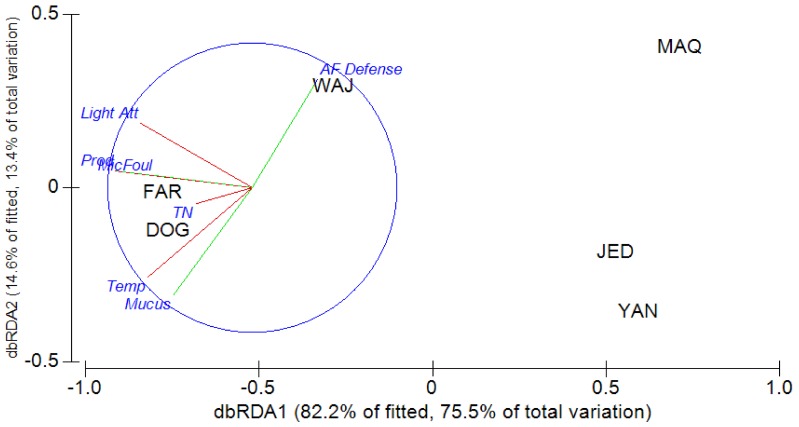
Distance-based redundancy analysis triplot. The site symbols represent the *P. verrucosa* populations distributed according to the similarities regarding the 3 “response” variables (productivity [“Prod”], mucus production [“Mucus”], antifouling defense strength [“AF Defense”]). Axis 1 relates negatively to light attenuation [“Light Att”], microbial fouling pressure [“MicFoul”] and temperature [“Temp”]. Axis 2 relates positively to light attenuation and microfouling, negatively to temperature. TN explains relatively little of the variance. Productivity relates positively to light and microfouling, mucus positively to temperature and defenses negatively to temperature. Stats results in S1–S5 Tables. (The 7^th^ pristine site, Mastura, is not shown because we have no mucus release data from that site.)

The anomalies plot (Fig. 5) gives a synoptic impression of the standardized large scale patterns of several coral traits at non-polluted stations with the amount of variation explained by the fitted regressions indicated in the plot. A sinusoidal decrease from north to south in defense strength (as mentioned), a slight linear increase for productivity (net photosynthesis) rates and a more pronounced linear increase of mucus release are apparent. Since along this N-S gradient several environmental variables (i.e. potential drivers of the biological response patterns) change simultaneously, we explored the complex relationships further using DISTLM (Fig. 6).The dbRDA triplot shows the distribution of the coral populations (distinguished by their site code) according to the similarity of the traits “Prod” (i.e. daily productivity), “Mucus” (i.e. daily mucus release) and “AF Defense” (the strength of chemical antisettlement activity in 2012). The populations are mostly separated along the first axis of the ordination plot. The ranked determination of the two axes by the four environmental factors is as follows (values in brackets are partial correlation coefficients). Axis 1: microbial fouling (−0.597)> temperature (−0.565)> light attenuation (−0.55). Axis 2: temperature (−0.727)> light attenuation (+0.665)> total N (+0.126). The first axis explains 82% of the fitted and 75% of the total variation. The second axis explains 15% of the fitted and 14% of the total variation. The third axis explains less than 4% and will be ignored. The first two axes, thus, cumulatively explain 89% of the total variation in the multivariate response of the 6 pristine *Pocillopora* populations investigated. The populations seem to cluster as southern (Far^7N^, Dog^6n^) and central-northern groups (Jed^5N^, Yan^3N^, Maq^1N^). The second most northern population, Waj^2N^, does not conform to this clustering. Mucus production follows the temperature pattern closely. Poductivity seems positively related to temperature and light attenuation. Chemical defenses and mucus production show opposing patterns. Subsequent multiple regressions reveal that light and temperature together explained 77% of the combined variance in the three response variables. Of these, chemical defense was not significantly related to any of the single environmental variables tested. Light attenuation explains 70% of the variance in productivity (p = 0.072) and temperature explains 86% of the variance in mucus production (p = 0.011).

## Discussion

In this study we described an environmental gradient along the Saudi Arabian Red Sea coast, assessed large scale variation in some coral traits, and screened for correlations among the abiotic and biotic traits.

Along the 2000 km of Saudi Red Sea coast the strength of antimicrofouling defenses in *P. verrucosa* exhibits a sinusoidal pattern with strongest defenses in the north, weak defenses in the south and an intermediate minimum followed by an intermediate maximum from north to south. Productivity and mucus release of the coral tend to increase linearly from north to south.

Along this coast, nutrients, total carbon, turbidity and temperature increased from north to south. Microfouling pressure (*sensu* microbial recruitment, i.e. settlement plus cell division plus detachment or mortality) is generally remarkably low with the exception of the southernmost sites. For comparison's sake, typical net microbial settlement in the open Pacific waters assessed by similar methods is about 2 orders of magnitude higher [58]. Since our microfouling data correlate significantly and positively with nutrients, but not with temperature, the overall low fouling pressure may reflect the oligotrophic nature of the Red Sea.

The observed large scale variability in chemical antimicrofouling patterns may be the result of a variety of causes or drivers. It could (1) be the product of stochastic fluctuations, (2) reflect genetic differences among regionally separate populations of the coral holobiont, (3) mirror differences in the fitness status of the populations sampled (e.g. regional to local impact of pollution or other stresses), (4) reflect variances in microfouling pressure or (5) be related to the variance in mucus release which, among other functions, may constitute a complementary antimicrofouling mechanism. Naturally, more than one of these drivers can be responsible for the observed pattern.

**Figure 7:**
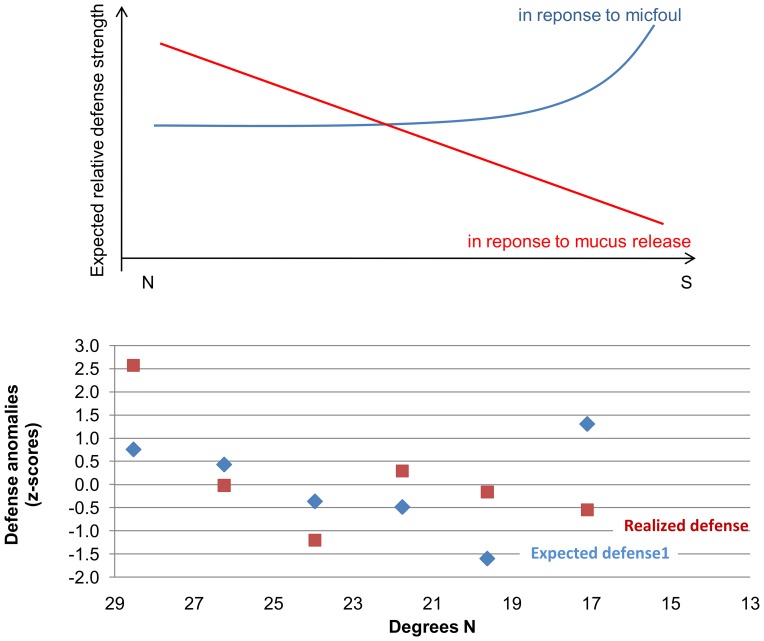
Expected versus realized chemical antimicrofouling response. Upper panel: Theoretical response of chemical antifouling defense to the large scale patterns of microfouling pressure and mucus release detected along the Saudi Arabian coast. Lower panel: Expected defense pattern calculated as z-scores of microfouling pressure (supposedly positively related to chemical defense) minus the z-scores of mucus release (supposedly negatively related to chemical defense) and the pattern of realized defense.

Stochasticity seems unlikely since sampling was replicated among genotypes and years within populations and still the pattern was consistent. No evidence for genetic differentiation at the regional scale was detected by Sawall et al. [55] and Sawall et al. (under review). Productivity (as a proxy for “fitness”) of the corals did not relate positively or negatively to defense strength. In contrast, supposedly polluting point sources (cities, industrial facilities) featured coral populations with weakened defenses. Higher microfouling pressure in the south could be expected to require, possibly even trigger, stronger defenses in the southern population. Interestingly, at this extreme site of the environmental gradient, the two potential antimicrofouling mechanisms, secondary metabolites (“chemical”) and mucus secretion showed opposing patterns. The former was particularly weak, the second particularly strong. The rate of mucus release could affect the strength of chemical antimicrofouling defense in two ways at least: (1) fast production and release of mucus would dilute the concentration of antimicrofouling compounds at the holobiont's surface and (2) given the cleansing role of mucus release, chemical antifouling might be less required in situations of fast mucus release. If chemical antifouling defense was determined by microfouling pressure (positively) and by mucus release (negatively), it should decrease from the north to the centre with a levelling out or a slight increase towards the southernmost sites. Fig. 7 shows the theoretical relationships in the upper panel and the expected (based on the combined anomalies of microbial fouling and mucus release) versus realized defense in the lower panel. While the realized chemical defense very roughly follows the expected trend, at the southernmost site it is much weaker than the expected level whereas it is higher than expected at the northernmost site. The opposing vectors of defense and mucus in the dbRDA hint at an anticorrelation of mucus release and chemical antimicrofouling. As a consequence, the *combined* defense (mucus plus secondary metabolites) might be assured everywhere along the coast gradually shifting in a southward direction from mostly a chemical to a mostly mechanical nature.

Mucus secretion at the coral surface is effective in removing both dead (seston) and living (bacteria, larvae) particles [31], while chemical defenses are only repelling (or killing) potential settlers. Consequently, mucus secretion might be the better defense alternative in the southern part of the Red Sea where particle densities were increased. It is, however, particularly costly to the producer [59]. Coral mucus isolated from a Caribbean *Acropora* species, presumably including its associated bacteria and the secondary metabolites by both bacteria and polyp, shows an antimicrobial activity towards a number of non-associated strains [36]. This mucus-associated chemical activity weakens when temperatures rise beyond a species specific threshold [24], [25].

Supposed stress on the scleractinian coral *Pocillopora verrucosa* increases from north to south [55] regarding summer temperature (exceeding 31°C in the south), particle load (i.e. shading) and microfouling pressure (more than 3x higher in the south). Even though our investigation was carried out in the thermally less demanding winter seasons, it illustrated that chemical defenses related negatively to temperature. Warmer temperatures bear the potential for driving a re-structuring of the microbial associated community changes in the physical properties of the mucus, for a reduction of the antimicrobial properties of the mucus and the polyp exudates, for the onset of virulence in associated bacteria, for enhanced prevalence of pathogenic bacteria, for heightened sensitivity of the polyps to infection and – maybe as sum responses – for enhanced coral diseases and bleaching [22], [24], [25], [33], [36]. High particle load also reduces light penetration, potentially leading to reduced productivity and further to reduced energy availability for defense. However, the shallow depth (3–5 m) at which our coral samples were taken makes light limitation by a southwardly increasing Kd (photosynthetic active radiation usually still >600 µE m^−2^ s^−1^ during mid-day) unlikely (e.g. [60]), The higher fouling pressure likely requires higher investment into defense (mechanical mucus sloughing or chemical antifouling agents).Despite this impressive list of presumed stresses increasing from north to south, increasing coral productivity in the same direction (in March at least) suggests that the corals fair well along the entire environmental gradient. So, if there is a stress gradient along the Saudi Arabian Red Sea coast with regard to the performance of reef corals it is compound and, probably, seasonally variable.

To conclude, we found major variation in biological traits of the holobiont *Pocillopora verrucosa* in a multi-factorial, latitudinal gradient along the 2000 km Saudi Arabian Red Sea coast. The proximity to (supposedly) polluting sources impacted chemical antimicrobial defense. Temperature, microbial fouling pressure and turbidity (light attenuation) explained much of the variation in the combined biological traits. While the variance in chemical defense could not be attributed to environmental factors, increasing temperature related to enhanced mucus production. Since mucus production and chemical defense showed somewhat opposing patterns, their combined antifouling effect seemed not jeopardized in any position along the environmental gradient. Further warming (together with increasing nutrient loads and coastal pollution) may shift defenses from chemical to mechanical (mucus) in all Red Sea populations. The latter is likely to be metabolically particularly expensive [59], and energy supply (irradiation) is not expected to increase with temperature. This may put a higher energetic strain on corals.

### Ethics Statement

The research permission was obtained from the Minister of Higher Education and the Ministry of Defense, Department of Marine Survey 273 in Saudi Arabia. The sampled reefs are not legally protected or part an MPA. Consequently, a special permit was not required for our work on these reefs. The Saudi Coastguard Authority issued sailing permits to the sites that included coral collection. *Pocillopora verrucosa* is listed as “least concern” on the ICUN Red List (http://www.iucnredlist.org). The CITES permission number is 11-SA-0197-PD.

## Supporting Information

S1 Table Distance based linear models results (DistLim) with all environmental variables and the compound response of the various coral populations (mucus, defense, productivity).
(DOCX)

S2 Table Distance based redundancy analysis with all environmental variables and the compound response of the various coral populations (mucus production, antifouling defense, productivity).
(DOCX)

S3 Table DistLim with only one response: Mucus release.
(DOCX)

S4 Table DistLim with only one response: Chemical Defense.
(DOCX)

S5 Table DistLim with only one response: Productivity.
(DOCX)
