# Supplementary material for: Large Scale Patterns of Antimicrofouling Defenses in the Hard Coral Pocillopora verrucosa in an Environmental Gradient along the Saudi Arabian Coast of the Red Sea
Source: PLoS One. 2014 Dec 8;9(12):e106573. doi: 10.1371/journal.pone.0106573 (PMC4259301; doi:10.1371/journal.pone.0106573)
Supplement: S1 Table — Distance based linear models results (DistLim) with all environmental variables and the compound response of the various coral populations (mucus, defense, productivity). (DOCX) [file pone.0106573.s001.docx]

Table S1: Distance based linear models results (DistLim) with all environmental variables and the compound response of the various coral populations (mucus, defense, productivity)

*Resemblance worksheet*

Name: Resem7_AllData_wBa

Data type: Distance

Selection: All

Transform: Square root

Resemblance: D1 Euclidean distance

*Predictor variables worksheet*

Name: EnvData_MicFoul

Data type: Other

Sample selection: All

Variable selection: All

Selection criterion: AICc

Selection procedure: Best

| *VARIABLES* |  |  |
| --- | --- | --- |
| 1 | Light Att | Trial |
| 2 | Temp | Trial |
| 3 | TN | Trial |
| 4 | MicFoul | Trial |
| Total SS(trace): 3.1735 |  |  |

| *MARGINAL TESTS* |  |  |
| --- | --- | --- |
| Variable | SS(trace) | Pseudo-F |
| P | Prop. |  |
| Light Att | 1.4933 | 3.5552 |
| 0.116 | 0.47056 |  |
| Temp | 1.427 | 3.268 |
| 0.148 | 0.44964 |  |
| TN | 0.44908 | 0.65933 |
| 0.491 | 0.14151 |  |
| MicFoul | 0.98728 | 1.8063 |
| 0.197 | 0.3111 |  |
| res.df: 4 |  |  |

*BEST SOLUTIONS*

BEST RESULT FOR EACH NUMBER OF VARIABLES

| AICc | R^2 | RSS | No.Vars | Selections |
| --- | --- | --- | --- | --- |
| 0.36289 | 0.47056 | 1.6802 | 1 | 1 |
| 5.1638 | 0.77742 | 0.70638 | 2 | 1,2 |
| 32.284 | 0.86227 | 0.43708 | 3 | 1,2,4 |
| Infinity | 0.9179 | 0.26055 | 4 | All |

*OVERALL BEST SOLUTIONS*

| AICc | R^2 | RSS | No.Vars | Selections |
| --- | --- | --- | --- | --- |
| 0.36289 | 0.47056 | 1.6802 | 1 | 1 |
| 0.59538 | 0.44964 | 1.7466 | 1 | 2 |
| 1.9426 | 0.3111 | 2.1863 | 1 | 4 |
| 3.2631 | 0.14151 | 2.7245 | 1 | 3 |
| 5.1638 | 0.77742 | 0.70638 | 2 | 1,2 |
| 9.2849 | 0.55763 | 1.4039 | 2 | 2,4 |
| 9.6805 | 0.52748 | 1.4996 | 2 | 1,3 |
| 9.866 | 0.51264 | 1.5466 | 2 | 1,4 |
| 10.222 | 0.48287 | 1.6411 | 2 | 2,3 |
| 11.652 | 0.34367 | 2.0829 | 2 | 3,4 |
