# Supplementary material for: Large Scale Patterns of Antimicrofouling Defenses in the Hard Coral Pocillopora verrucosa in an Environmental Gradient along the Saudi Arabian Coast of the Red Sea
Source: PLoS One. 2014 Dec 8;9(12):e106573. doi: 10.1371/journal.pone.0106573 (PMC4259301; doi:10.1371/journal.pone.0106573)
Supplement: S2 Table — Distance based redundancy analysis with all environmental variables and the compound response of the various coral populations (mucus production, antifouling defense, productivity). (DOCX) [file pone.0106573.s002.docx]

Table S2: Distance based redundancy analysis with all environmental variables and the compound response of the various coral populations (mucus production, antifouling defense, productivity)

*Resemblance worksheet*

Name: Resem7_AllData_wBa

Data type: Distance

Selection: All

Transform: Square root

Resemblance: D1 Euclidean distance

*Predictor variables worksheet*

Name: EnvData_MicFoul

Data type: Other

Sample selection: All

Variable selection: All

*Percentage of variation explained by individual axes*

|  | %explainedvariation | %explainedvariation |
| --- | --- | --- |
|  | outoffittedmodel | outoftotalvariation |
| Axis | Individual ; Cumulative | Individual ; Cumulative |
| 1 | 82.21 ; 82.21 | 75.46 ; 75.46 |
| 2 | 14.59 ; 96.8 | 13.39 ; 88.85 |
| 3 | 3.2 ; 100 | 2.94 ; 91.79 |

*dbRDA coordinate scores*

| Sample | dbRDA1 | dbRDA2 | dbRDA3 |
| --- | --- | --- | --- |
| MAQ | 0.71039 | 0.40075 | 9.2472E-2 |
| WAJ | -0.28634 | 0.28929 | -0.18005 |
| YAN | 0.59045 | -0.35914 | -0.14797 |
| JED | 0.52449 | -0.18822 | 0.11613 |
| DOG | -0.76161 | -0.12672 | 0.12958 |
| FAR | -0.77738 | -1.5965E-2 | -1.0166E-2 |

*Relationships between dbRDA coordinate axes and orthonormal X variables*

*(multiple partial correlations)*

| Variable | dbRDA1 | dbRDA2 | dbRDA3 |
| --- | --- | --- | --- |
| LightAtt | -0.546 | 0.665 | 0.164 |
| Temp | -0.565 | -0.727 | 0.285 |
| TN | 0.164 | 0.126 | 0.930 |
| MicFoul | -0.597 | 0.114 | -0.164 |

*Weights*

*(Coefficients for linear combinations of X's in the formation of dbRDA coordinates)*

| Variable | dbRDA1 | dbRDA2 | dbRDA3 |
| --- | --- | --- | --- |
| LightAtt | -20.214 | 10.504 | 1.3355 |
| Temp | -0.2116 | -0.10714 | -5.8414E-3 |
| TN | -4.8076E-2 | 0.29049 | 0.23949 |
| MicFoul | 5.1945E-2 | -3.7199E-2 | -1.7718E-2 |
