# Supplementary material for: Large Scale Patterns of Antimicrofouling Defenses in the Hard Coral Pocillopora verrucosa in an Environmental Gradient along the Saudi Arabian Coast of the Red Sea
Source: PLoS One. 2014 Dec 8;9(12):e106573. doi: 10.1371/journal.pone.0106573 (PMC4259301; doi:10.1371/journal.pone.0106573)
Supplement: S3 Table — DistLim with only one response: Mucus release. (DOCX) [file pone.0106573.s003.docx]

Table S3: DistLim with only one response: Mucus release

*Resemblance worksheet*

Name: Resem6_Response-Mucus

Data type: Distance

Selection: All

Transform: Square root

Resemblance: D1 Euclidean distance

*Predictor variables worksheet*

Name: EnvData_MicFoul

Data type: Other

Sample selection: All

Variable selection: All

Selection criterion: AICc

Selection procedure: Best

*VARIABLES*

1 LightAtt Trial

2 Temp Trial

3 TN Trial

4 MicFoul Trial

Total SS(trace): 0.44874

*MARGINAL TESTS*

| Variable | SS(trace) | Pseudo-F | P | Prop. |
| --- | --- | --- | --- | --- |
| LightAtt | 3.2458E-4 | 2.8954E-3 | 0.951 | 7.2332E-4 |
| Temp | 0.3878 | 25.454 | 0.008 | 0.8642 |
| TN | 0.11146 | 1.3219 | 0.335 | 0.24839 |
| MicFoul | 3.6295E-2 | 0.352 | 0.671 | 8.0881E-2 |

res.df: 4

NO STARTING TERMS

*BEST SOLUTIONS*

BEST RESULT FOR EACH NUMBER OF VARIABLES

| AICc | R^2 | RSS | No.Vars | Selections |
| --- | --- | --- | --- | --- |
| -19.538 | 0.8642 | 6.0941E-2 | 1 | 2 |
| -11.923 | 0.90874 | 4.0951E-2 | 2 | 1,2 |
| 17.58 | 0.916 | 3.7696E-2 | 3 | 1-3 |
| Infinity | 0.92033 | 3.5752E-2 | 4 | All |

*OVERALL BEST SOLUTIONS*

| AICc | R^2 | RSS | No.Vars | Selections |
| --- | --- | --- | --- | --- |
| -19.538 | 0.8642 | 6.0941E-2 | 1 | 2 |
| -11.923 | 0.90874 | 4.0951E-2 | 2 | 1,2 |
| -11.149 | 0.89617 | 4.6592E-2 | 2 | 2,4 |
| -10.206 | 0.8785 | 5.452E-2 | 2 | 2,3 |
| -9.2717 | 0.24839 | 0.33728 | 1 | 3 |
| -8.0645 | 8.0881E-2 | 0.41244 | 1 | 4 |
| -7.5628 | 7.2332E-4 | 0.44841 | 1 | 1 |
| 0.61253 | 0.26276 | 0.33083 | 2 | 1,3 |
| 0.71945 | 0.2495 | 0.33678 | 2 | 3,4 |
| 0.8761 | 0.22965 | 0.34569 | 2 | 1,4 |
