# Supplementary material for: Large Scale Patterns of Antimicrofouling Defenses in the Hard Coral Pocillopora verrucosa in an Environmental Gradient along the Saudi Arabian Coast of the Red Sea
Source: PLoS One. 2014 Dec 8;9(12):e106573. doi: 10.1371/journal.pone.0106573 (PMC4259301; doi:10.1371/journal.pone.0106573)
Supplement: S4 Table — DistLim with only one response: Chemical Defense. (DOCX) [file pone.0106573.s004.docx]

Table S4: DistLim with only one response: Chemical Defense

DistLM

Distance based linear models

*Resemblance worksheet*

Name: Resem7_Response-defense(wBa)

Data type: Distance

Selection: All

Transform: Square root

Resemblance: D1 Euclidean distance

*Predictor variables worksheet*

Name: EnvData_MicFoul

Data type: Other

Sample selection: All

Variable selection: All

Selection criterion: AICc

Selection procedure: Best

*VARIABLES*

1 LightAtt Trial

2 Temp Trial

3 TN Trial

4 MicFoul Trial

Total SS(trace): 0.26163

*MARGINAL TESTS*

| Variable | SS(trace) | Pseudo-F | P | Prop. |
| --- | --- | --- | --- | --- |
| LightAtt | 7.4819E-3 | 0.11776 | 0.782 | 2.8597E-2 |
| Temp | 0.10454 | 2.6617 | 0.165 | 0.39955 |
| TN | 1.2291E-3 | 1.888E-2 | 0.874 | 4.6979E-3 |
| MicFoul | 2.254E-2 | 0.37709 | 0.421 | 8.6151E-2 |
| res.df: 4 | | | | |

NO STARTING TERMS

*BEST SOLUTIONS*

BEST RESULT FOR EACH NUMBER OF VARIABLES

| AICc | R^2 | RSS | No.Vars | Selections |
| --- | --- | --- | --- | --- |
| -13.856 | 0.39955 | 0.1571 | 1 | 2 |
| -9.6248 | 0.77043 | 6.0062E-2 | 2 | 2,3 |
| 16.9 | 0.87136 | 3.3657E-2 | 3 | 2-4 |
| Infinity | 0.9872 | 3.3496E-3 | 4 | All |

*OVERALL BEST SOLUTIONS*

| AICc | R^2 | RSS | No.Vars | Selections |
| --- | --- | --- | --- | --- |
| -13.856 | 0.39955 | 0.1571 | 1 | 2 |
| -11.336 | 8.6151E-2 | 0.23909 | 1 | 4 |
| -10.97 | 2.8597E-2 | 0.25415 | 1 | 1 |
| -10.824 | 4.6979E-3 | 0.2604 | 1 | 3 |
| -9.6248 | 0.77043 | 6.0062E-2 | 2 | 2,3 |
| -3.8575 | 0.3997 | 0.15706 | 2 | 1,2 |
| -3.8566 | 0.39961 | 0.15708 | 2 | 2,4 |
| -2.0557 | 0.18944 | 0.21207 | 2 | 3,4 |
| -1.4658 | 0.1057 | 0.23398 | 2 | 1,4 |
| -1.0617 | 4.3405E-2 | 0.25027 | 2 | 1,3 |
