# Supplementary material for: Large Scale Patterns of Antimicrofouling Defenses in the Hard Coral Pocillopora verrucosa in an Environmental Gradient along the Saudi Arabian Coast of the Red Sea
Source: PLoS One. 2014 Dec 8;9(12):e106573. doi: 10.1371/journal.pone.0106573 (PMC4259301; doi:10.1371/journal.pone.0106573)
Supplement: S5 Table — DistLim with only one response: Productivity. (DOCX) [file pone.0106573.s005.docx]

Table S5: DistLim with only one response: Productivity

*Resemblance worksheet*

Name: Resem8_Response-Productivity

Data type: Distance

Selection: All

Transform: Square root

Resemblance: D1 Euclidean distance

*Predictor variables worksheet*

Name: EnvData_MicFoul

Data type: Other

Sample selection: All

Variable selection: All

Selection criterion: AICc

Selection procedure: Best

*VARIABLES*

1 LightAtt Trial

2 Temp Trial

3 TN Trial

4 MicFoul Trial

Total SS(trace): 2.4632

*MARGINAL TESTS*

| Variable | SS(trace) | Pseudo-F | P | Prop. |
| --- | --- | --- | --- | --- |
| Light Att | 1.4855 | 6.0781 | 0.065 | 0.6031 |
| Temp | 0.93462 | 2.4458 | 0.199 | 0.37944 |
| TN | 0.33638 | 0.63266 | 0.417 | 0.13657 |
| MicFoul | 0.92845 | 2.4199 | 0.075 | 0.37693 |
| res.df: 4 | | | | |

NO STARTING TERMS

*BEST SOLUTIONS*

BEST RESULT FOR EACH NUMBER OF VARIABLES

| AICc | R^2 | RSS | No.Vars | Selections |
| --- | --- | --- | --- | --- |
| -2.8863 | 0.6031 | 0.97763 | 1 | 1 |
| 3.1901 | 0.79361 | 0.50837 | 2 | 1,2 |
| 28.671 | 0.90282 | 0.23937 | 3 | 1,2,4 |
| Infinity | 0.91009 | 0.22145 | 4 | All |

*OVERALL BEST SOLUTIONS*

| AICc | R^2 | RSS | No.Vars | Selections |
| --- | --- | --- | --- | --- |
| -2.8863 | 0.6031 | 0.97763 | 1 | 1 |
| -0.20468 | 0.37944 | 1.5285 | 1 | 2 |
| -0.18049 | 0.37693 | 1.5347 | 1 | 4 |
| 1.7771 | 0.13657 | 2.1268 | 1 | 3 |
| 3.1901 | 0.79361 | 0.50837 | 2 | 1,2 |
| 6.7391 | 0.62712 | 0.91846 | 2 | 1,3 |
| 7.048 | 0.60742 | 0.96698 | 2 | 1,4 |
| 8.3444 | 0.51274 | 1.2002 | 2 | 2,4 |
| 9.7875 | 0.38024 | 1.5266 | 2 | 2,3 |
| 9.8169 | 0.37721 | 1.534 | 2 | 3,4 |
